# The difference between the effectiveness of body-weight-supported treadmill training combined with functional electrical stimulation and sole body-weight-supported treadmill training for improving gait parameters in stroke patients: A systematic review and meta-analysis

**DOI:** 10.3389/fneur.2022.1003723

**Published:** 2022-11-09

**Authors:** Jiaqi Wang, Liangyu Zhao, Yan Gao, Chenchen Liu, Xiaosheng Dong, Xiqian He

**Affiliations:** ^1^School of Physical Education, Shandong University, Jinan, China; ^2^School of Medical Information Engineering, Jining Medical University, Jining, China; ^3^Jining No.1 People's Hospital, Jining, China

**Keywords:** stroke patients, functional electrical stimulation, body weight support, treadmill training, randomized controlled trials

## Abstract

**Background:**

Body-weight-supported treadmill training (BWSTT) combined with functional electrical stimulation (FES) is considered an effective intervention method to improve gait parameters in stroke patients. In this article, we compared the effect of BWSTT combined with FES and BWSTT only on gait parameters in stroke patients.

**Methods:**

Two researchers searched for literature published before January 5, 2021, in seven Chinese and English databases including PubMed, Web of Science, Cochrane Library, Ovid, CNKI, Wanfang Data, and VIP. Meta-analysis was then performed on various data collected, namely, 10 Meters Walking Test (10MWT), gait speed, Fugl-Meyer Assessment (FMA), Berg Balance Scale (BBS), Modified Barthel Index (MBI), Comprehensive Spasticity Scale (CSS), Functional Ambulation Category (FAC), and Ankle Range of Motion (AROM).

**Results:**

A total of 14 studies were included in the meta-analysis, in which 945 stroke patients participated. In these 14 studies, the participants were randomly divided into a test group and a control group. The test group received BWSTT combined with FES, while the control group received BWSTT only. Meta-analysis showed that when compared to BWSTT, BWSTT combined with FES had a better effect on FAC, AROM, 10MWT, CSS, MBI, FMA, gait speed, and BBS of stroke patients. However, the effect of BWSTT combined with FES on BBS was not significant in the medium exercise group when compared to that of BWSTT. Also, the effect of BWSTT combined with FES on gait speed was not significant in the large exercise group when compared to that of BWSTT only.

**Conclusion:**

BWSTT combined with FES is more effective than BWSTT only for improving gait parameters in stroke patients.

**Systematic review registration:**

https://www.crd.york.ac.uk/prospero/#recordDetails, CRD42022299636.

## Introduction

Stroke is a kind of cerebrovascular disease that is characterized by high mortality and high disability, and poses a serious health risk to people (1). Although the risk of stroke was perceived to be relatively less for young people, the number has started increasing in recent times. In China, 50% of stroke patients were under 65 years of age, and nearly 15% of stroke patients were under 45 years of age (2). Stroke affected not only the health of individuals, but also the activities of their daily life (3). The health complications that occurred after the incidence of stroke could be very debilitating, such as decreased proprioception (4), impaired balance (5), altered gait (6), and decreased muscle strength, coordination, and dexterity (7, 8). Such complications seriously affected the general health status of patients and inhibit them from returning to society (9, 10). Hence, it is important to find effective ways to promote the rehabilitation of stroke patients and reduce complications after stroke. Improving the gait parameters in stroke patients and helping them return to normal life as soon as possible is one of the main goals of stroke patients' rehabilitation.

Body-weight-supported treadmill training (BWSTT) combined with functional electrical stimulation (FES) is an effective method for improving gait parameters in stroke patients. BWSTT is a functional training method that is based on the reorganization of brain function and neuroplasticity (11). It involves using suspension devices to reduce the load on patients' lower limbs and using electric treadmills to enable patients' lower limbs to carry out repetitive and rhythmic gait cycle exercises (12). FES is aimed to improve the structural integrity of lower motor neurons in patients with central paralysis. It stimulates nerves and muscles using low-frequency electrical pulses to produce immediate functional activity. This treatment has a lasting and positive effect on patients' gait, posture, and voluntary control of movement. It also helps patients relearn and reorganize the function of paralyzed limbs (13).

At present, BWSTT combined with FES is being widely used in clinical practice to treat stroke patients, as this combined intervention has been proven to have a good effect on the gait parameters of the patients. Hesse et al. (14) studied the positive effects of BWSTT combined with FES on the gait functions of stroke patients with hemiplegia. They reported that the combined intervention effectively improved leg muscle strength, stride length, stride frequency, gait speed, and gait pattern in patients with hemiplegia (14). Lindquist et al. (15) found that BWSTT combined with FES could improve walking function and lower limb voluntary control in patients with chronic hemiplegia. Meanwhile, Ng et al. (16) found that FES combined with weight-loss gait training significantly improved FAC and gait speed of stroke patients.

Research showed that BWSTT combined with FES could effectively reduce lower limb spasms, droop, varus, and other adverse conditions in stroke patients (17). However, it had also been shown that BWSTT was more better than BWSTT combined with FES for improving the gait parameters in stroke patients (16). Therefore, whether BWSTT combined with FES has a better effect on improving gait parameters in stroke patients than BWSTT only is worth further study. At present, there is no systematic meta-analysis to compare the effect of BWSTT combined with FES and BWSTT only on the gait parameters of stroke patients. Therefore, this review aims to systematically compare the effects of BWSTT combined with FES and BWSTT only in improving gait parameters in stroke patients.

## Methods

### Agreement registration

To study the effect of BWSTT combined with FES on the gait parameters of stroke patients, we conducted a meta-analysis and systematic evaluation according to the Preferred Reporting Items for Systematic Reviews and Meta-Analyses 2020 (PRISMA) statement (18), which has been registered in PROSPERO (CRD 42022299636).

### Data sources and search strategy

Two researchers independently searched electronic resource databases, including Web of Science, PubMed, Cochrane Library, Ovid, CNKI, WanFang, and VIP, for randomized controlled trials related to the subject. The search covered all data up to January 5, 2021, and included a bibliography of the articles to prevent omission. A target word (stroke) and an intervention word (weight support, treadmill training, and FES) were searched for in all the databases. Based on multiple previous studies, all searches used a topical search and a related-term search. The search terms included gait training, walking training, treadmill training, weight support treadmill training, stroke, cerebrovascular disease, hemiplegia, SAH, functional electrical stimulation, FES, etc. The search strategy for all the databases is presented in Appendix 1.

### Inclusion and exclusion criteria

The basic inclusion criterion was that all randomized controlled trials published in Chinese and English, without restriction on the age or sex of stroke patients should be included. The detailed inclusion criteria were (a) studies of stroke patients diagnosed with cerebral infarction or cerebral hemorrhage identified through Computer Tomography (CT) or Magnetic Resonance Imaging (MRI); (b) studies in which the participants were in stable condition, conscious, and able to cooperate to complete all assessments and receive motor instructions; (c) studies in which the patients did not have various osteoarticular or neuromuscular diseases that affect walking ability; (d) studies in which BWSTT and FES were used as the main intervention method for stroke exercise in the test group; (e) studies in which only BWSTT was used as the main intervention method for stroke exercise in the control group; (f) relevant studies that included relevant indicators of gait parameters.

Exclusion criteria were: (a) studies in which the main intervention method was not BWSTT combined with FES in the test group; (b) studies in which the main intervention method was not BWSTT only in the control group; (c) repeated articles or articles with incomplete data; (d) studies published in languages other than English or Chinese.

### Filtering and data extraction

Two researchers (JQW and LYZ) extracted and imported the searched articles into Endnote X9, and deleted duplicated articles. They initially screened the articles based on the titles and abstracts, and then screened them again based on the main text. When they had differing opinions on the inclusion of a paper during the screening process, they decided whether to include the article or not by consulting with a third researcher (YG). The descriptive information present in the articles was extracted and incorporated into the database by the third researcher. This included the article title, country, year of publication, name of the first author, sample size (number of participants in the experiment group and the control group), gender of the sample size, intervention methods of the experiment group and the control group, and the changes (mean value and standard deviation) in the research parameters. All this information was recorded in an Excel spreadsheet and verified by all three researchers.

### Risk of bias assessment

Using the Cochrane Risk of Bias tool present in the Cochrane Manual and Review Manager 5.4.1 software (19), two researchers (JQW and LYZ) strictly assessed the articles based on seven criteria. These were, “how was the random sequence generated?”, “did the researcher responsible for the allocation strictly allocate the result of random numbers?”, “were the participants and trial researchers double-blinded?”, “was the result assessor blinded?”, “was the result data complete?”, “were the positive results selectively reported?”, and “were there other factors that may cause bias?” The articles were assessed using the Review Manager 5.4.1 software. Disagreements were resolved by the third researcher (YG).

### Statistical analysis

Review Manager 5.4.1 and Stata 17.0 were used for the meta-analysis. As the articles had the same continuous outcome variables and the same units of measurement, weighted mean difference was calculated. In case the mean and standard deviations of the studied indicators were not published, they were estimated based on pre- and post-intervention values according to the Cochrane Handbook for Systematic Reviews of Interventions. Owing to the differences in the study designs and study populations, the random effects model was used in the meta-analysis, assuming that the effect sizes were different among studies. Q statistic and *I*^2^ statistic were used to test for heterogeneity. When the heterogeneity was *p* > 0.1 and *I*^2^ > 50%, subgroup analysis was conducted based on the total exercise volume. Since the intervention in the test group was BWSTT combined with FES, the differences in the exercise intensity among the selected studies were not considered in this study. The total exercise volume was determined based on the total exercise time. The included studies were divided into three subgroups based on the total exercise volume—small exercise group (<50,000 s), medium exercise group (50,000–100,000 s), and large exercise group (more than 100,000 s). The total exercise volume (in seconds) was equal to the intervention time of BWSTT combined with FES per day multiplied by the intervention days of BWSTT combined with FES per week multiplied by the total number of weeks of combined intervention (one month was considered to have 4 weeks). If the between-group heterogeneity was too large or the number of articles was too small for subgroup analysis, descriptive analysis was conducted. Sensitivity analysis was conducted if necessary, and funnel plot and Egger's test were used to assess possible publication bias. The trim and filling method was used to correct the possible bias, and the cause of the publication bias was identified through meta-regression.

## Results

### Included studies and main characteristics

**Figure 1:**
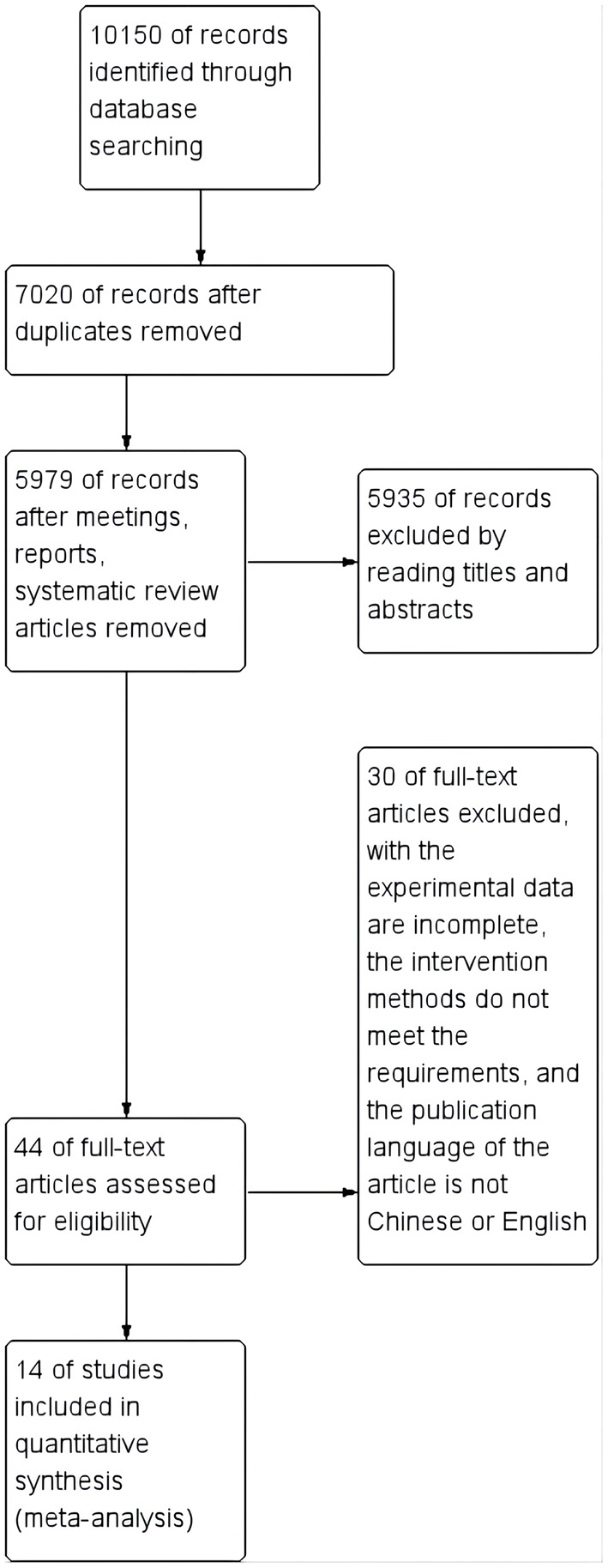
Flow diagram of study selection and identification.

A total of 10,150 articles were retrieved from various Chinese and foreign language databases. Three thousand one hundred thirty repetitive articles were removed using Document Manager. Five Thousand nine hundred thirty-five irrelevant articles were removed by reading titles and abstracts, and 1,041 relevant articles were identified by reading through relevant meta-analysis literature and systematic reviews. Finally, 44 potentially eligible articles were selected. Out of these, unqualified articles were excluded by reading full texts, and 14 controlled experiments were selected for the study (Figure 1).

A total of 945 subjects were included in the 14 selected articles. The basic characteristics of the included studies are presented in Table 1. The studies were conducted in China, Korea, and America. The sample size ranged from 29 to 180, and the duration of intervention ranged from 3 to 12 weeks. The frequency of intervention was four to six times per week, and each intervention lasted about 1,200–5,400 s. The test group received BWSTT combined with FES, and the control group received BWSTT only.

**Table 1:** Characteristics and quality assessments of the included trials.

**Studies**	**Patients (intervention/ control)**	**Age ±SD (intervention/ control)**	**Treatment parameters**	**Test group intervention**	**Control group intervention**	**Outcomes indicators**
Cheng et al. (20) (China)	30/30	56.22 ± 8.13 (T^a^) 52.19 ± 9.17 (C^b^)	120.0 s/day 5–6 days/week 3 weeks	Body Weight Support Treadmill Training (BWSTT) + Functional Electrical Stimulation (FES)	Body Weight Support Treadmill Training (BWSTT)	Ankle Range of Motion (AROM), Gait Speed, Functional Ambulation Category (FAC), Fugl-Meyer Assessment (FMA), Modified Barthel Index (MBI)
Cheng et al. (21) (China)	30/30	53.2 ± 6.2 (T) 52.6 ± 7.4 (C)	120.0 s/day 6 days/week 3 weeks	Body Weight Support Treadmill Training (BWSTT) + Functional Electrical Stimulation (FES)	Body Weight Support Treadmill Training (BWSTT)	Ankle Range of Motion (AROM), Gait Speed, Functional Ambulation Category (FAC), Fugl-Meyer Assessment (FMA), Modified Barthel Index (MBI)
	30/30	54.2 ± 8.3 (T) 52.6 ± 7.4 (C)	Body Weight Support Treadmill Training (BWSTT): 120.0 s/day 6 days/week 3 weeks Functional Electrical Stimulation (FES): 120.0 s/day 6 days/week 3 weeks	Body Weight Support Treadmill Training (BWSTT) + Functional Electrical Stimulation (FES)		
Chen et al. (12) (China)	21/20	59.5 ± 12.3 (T) 58.9 ± 10.5 (C)	180.0 s/day 6 days/week 8 weeks	Body Weight Support Treadmill Training (BWSTT) + Functional Electrical Stimulation (FES)	Body Weight Support Treadmill Training (BWSTT)	Comprehensive Spasticity Scale (CSS), Functional Ambulation Category (FAC), Berg Balance Scale (BBS)
Lee et al. (22) (Korea)	15/15	52.47 ± 9.41 (T) 56.73 ± 7.24 (C)	180.0 s/day 5 days/week 4 weeks	Body Weight Support Treadmill Training (BWSTT) + Functional Electrical Stimulation (FES)	Body Weight Support Treadmill Training (BWSTT)	Berg Balance Scale (BBS), Gait Speed
Daly et al. (23) (America)	20/34	59 (T) 62 (C)	540.0 s/ session 4 sessions /week 12 weeks	Body Weight Support Treadmill Training (BWSTT) + Functional Electrical Stimulation (FES)	Body Weight Support Treadmill Training (BWSTT)	Gait Speed
Li et al. (24) (China)	43/43	53 ± 4 (T) 54 ± 5 (C)	180.0 s/day 5 days/week 8 weeks	Body Weight Support Treadmill Training (BWSTT) + Functional Electrical Stimulation (FES)	Body Weight Support Treadmill Training (BWSTT)	10 M Walking Test (10MWT), Fugl-Meyer Assessment (FMA), Comprehensive Spasticity Scale (CSS)
Liu et al. (25) (China)	25/25	62.3 ± 11.8 (T) 62.1 ± 11.3 (C)	Body Weight Support Treadmill Training (BWSTT):	Body Weight Support Treadmill Training (BWSTT)+ Functional	Body Weight Support Treadmill Training (BWSTT)	10 M Walking Test (10MWT), Fugl-Meyer Assessment (FMA)
			360.0 s/day 5 days/week 4 weeks Functional Electrical Stimulation (FES): 240.0 s/day 5 days/week 4 weeks	Electrical Stimulation (FES)		
Li et al. (17) (China)	21/20	56.11 ± 8.1 (T) 54.30 ± 9.3 (C)	180.0 s/day 5 days/week 8 weeks	Body Weight Support Treadmill Training (BWSTT)+ Functional Electrical Stimulation (FES)	Body Weight Support Treadmill Training (BWSTT)	10 M Walking Test (10MWT), Fugl-Meyer Assessment (FMA), Comprehensive Spasticity Scale (CSS), Ankle Range of Motion (AROM)
Long et al. (26) (China)	50/50	52.63 ± 5.57 (T) 51.47 ± 4.64 (C)	180.0 s/day 5 days/week 3 weeks	Body Weight Support Treadmill Training (BWSTT) + Functional Electrical Stimulation (FES)	Body Weight Support Treadmill Training (BWSTT)	Modified Barthel Index (MBI)
Ng et al. (16) (China)	16/17	62 ± 10 (T) 66.6 ± 11.3 (C)	20 mins/day 5 days/week 4 weeks	Body Weight Support Treadmill Training (BWSTT) + Functional Electrical Stimulation (FES)	Body Weight Support Treadmill Training (BWSTT)	Functional Ambulation Category (FAC), Berg Balance Scale (BBS)
Cho et al. (27) (Korea)	10/11	57 ± 9.1 (T) 57.8 ± 7.9 (C)	30 mins/day 5 days/week 4 weeks	Body Weight Support Treadmill Training (BWSTT) + Functional Electrical Stimulation (FES)	Body Weight Support Treadmill Training (BWSTT)	Berg Balance Scale (BBS), Gait Speed
	10/11	53.3 ± 9.2 (T) 57.8 ± 7.9 (C)	30 mins/day 5 days/week 4 weeks	Body Weight Support Treadmill Training (BWSTT) + Functional Electrical Stimulation (FES)	Body Weight Support Treadmill Training (BWSTT)	
Qie et al. (28) (China)	16/13	54 ± 8.83 (T) 54.46 ± 7.39 (C)	180.0 s/day 5 days/week 4 weeks	Body Weight Support Treadmill Training (BWSTT) + Functional Electrical Stimulation (FES)	Body Weight Support Treadmill Training (BWSTT)	10 M Walking Test (10MWT), Fugl-Meyer Assessment (FMA), Berg Balance Scale (BBS)
Qin (29) (China)	60/60	52.49 ± 5.46 (T) 51.48 ± 5.38 (C)	180.0 s/day 5 days/week 8 weeks	Body Weight Support Treadmill Training (BWSTT) + Functional Electrical Stimulation (FES)	Body Weight Support Treadmill Training (BWSTT)	Modified Barthel Index (MBI)
Bao et al. (30) (China)	90/90	61.7 ± 7.09 (T) 60.8 ± 6.35 (C)	540.0 s/day 5 days/week 8 weeks	Body Weight Support Treadmill Training (BWSTT) + Functional Electrical Stimulation (FES)	Body Weight Support Treadmill Training (BWSTT)	Gait Speed, 10 M Walking Test (10MWT), Fugl-Meyer Assessment (FMA), Comprehensive Spasticity Scale (CSS)

^a^T indicates test group.

^b^C indicates control group.

Mins, minutes; SD, standard deviation.

### Risk of bias in included studies

**Figure 2:**
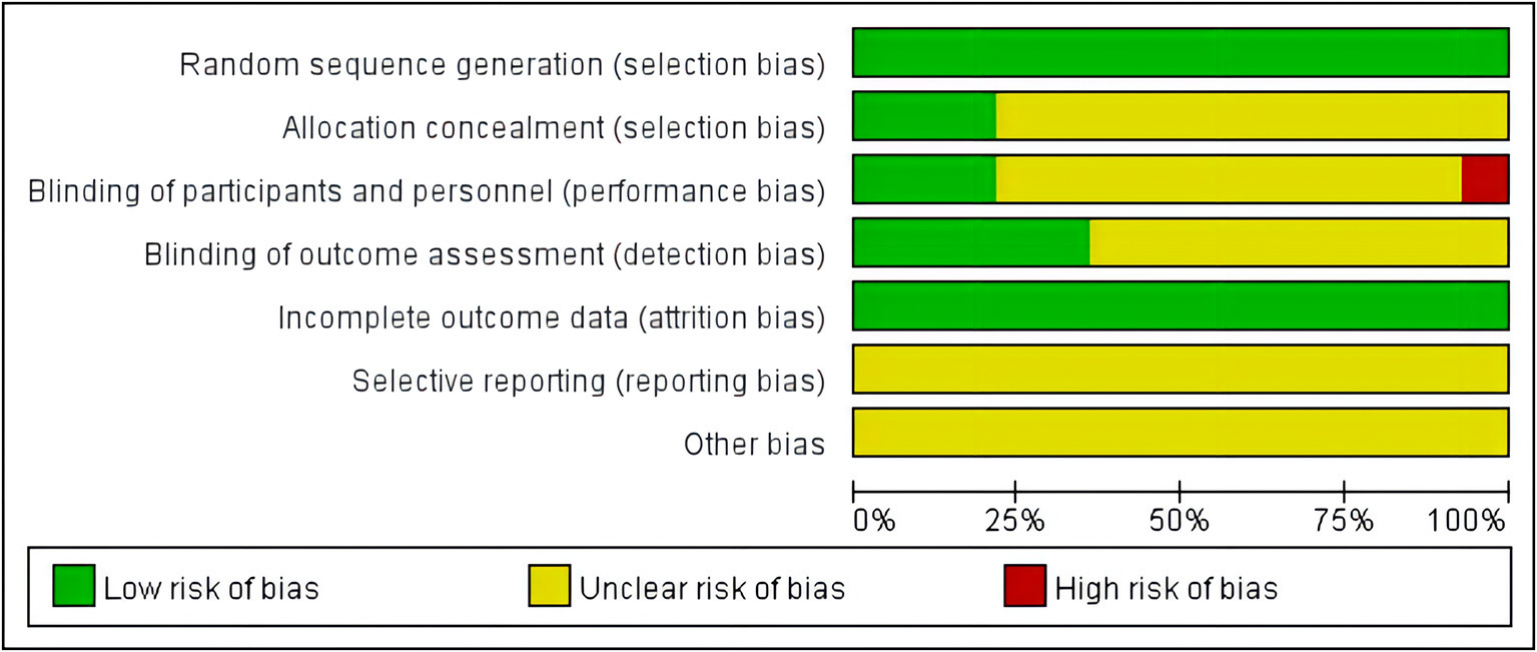
Risk of bias graph: judgements about each risk of bias item presented as percentages across all included studies.

**Figure 3:**
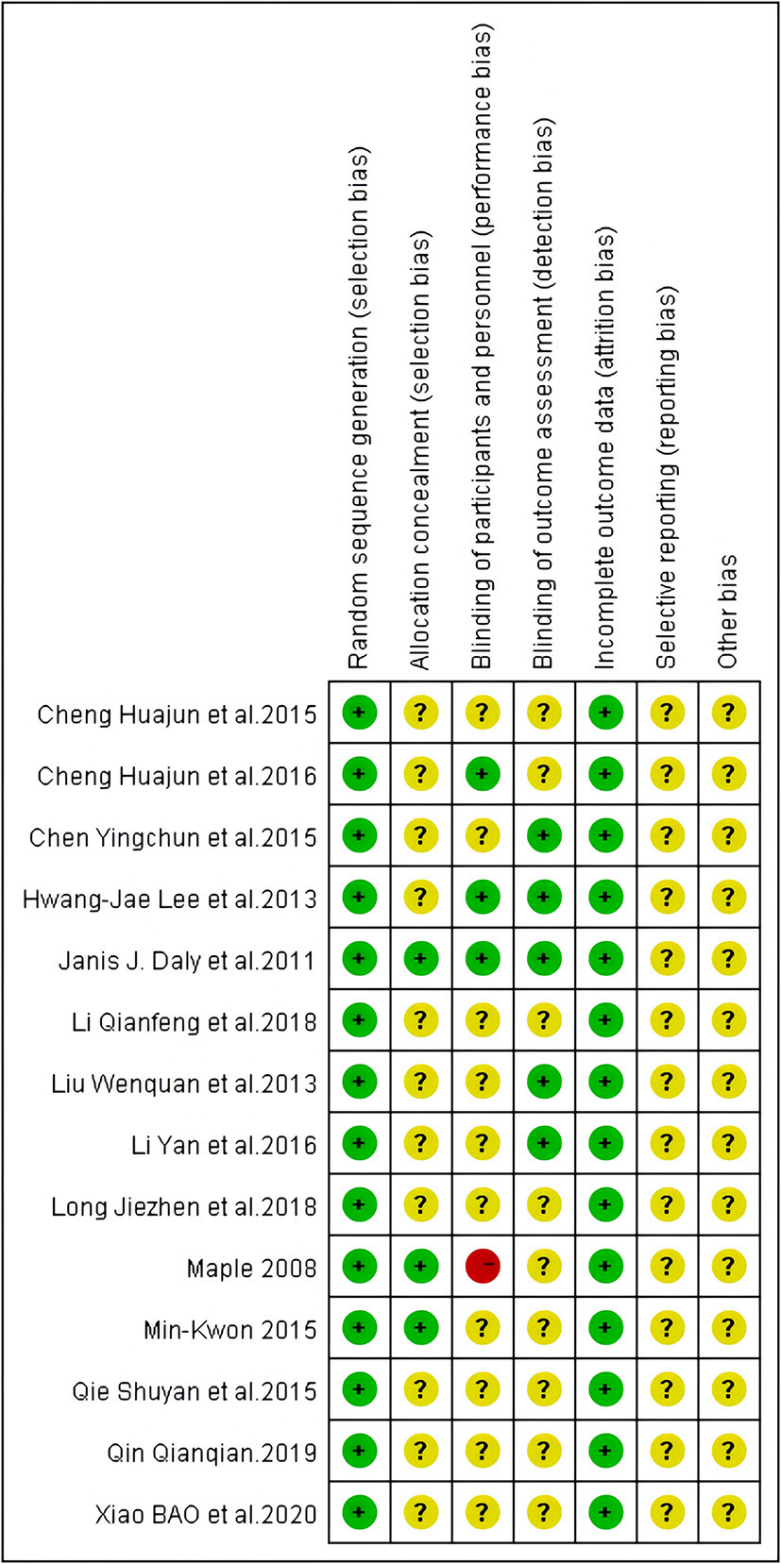
Risk of bias summary: judgements about each risk of bias item for each included study.

Two independent reviewers (JQW and LYZ) conducted the risk of bias assessment. In total, 14 studies had adequate random sequence generation. With respect to “selection bias,” 3 studies had adequate allocation concealment, while 11 did not give enough information. With respect to “performance bias,” 4 studies blinded participants and researchers, while 10 did not give enough information. Overall, 5 studies reported a blinded outcome assessment, while 9 did not give enough information. Fourteen studies provided complete outcome data. For all the studies, bias reporting was not mentioned because the protocol was not described. With respect to “other bias,', 14 studies did not give enough information (Figures 2, 3).

### Effect of interventions: Analyses by subgroups

The purpose of this study was to compare the difference between the effects of BWSTT combined with FES and BWSTT only in improving gait parameters in stroke patients. Gait parameters mainly included data from 10 Meters Walking Test (10MWT), Gait Speed, Fugl-Meyer Assessment (FMA), Berg Balance Scale (BBS), Modified Barthel Index (MBI), Comprehensive Spasticity Scale (CSS), Functional Ambulation Category (FAC), and Ankle Range of Motion (AROM) indicators. Meta-analysis and subgroup analysis were conducted for all research indicators.

Exercise load was used as a classification index for the subgroup analysis, and the total intervention time of BWSTT combined with FES was used as a measurement standard for exercise load. The exercise groups were classified as follows: small exercise group with an intervention time of < 50,000 s, medium exercise group with an intervention time of 50,000–100,000 s, and large exercise group with an intervention time of more than 100,000 s. Subgroup analysis based on this classification could better examine the differences between the effects of BWSTT combined with FES and that of BWSTT only, and further explore whether exercise load has any effect on gait parameters in stroke patients, and if so, which effect is better.

**Figure 4:**
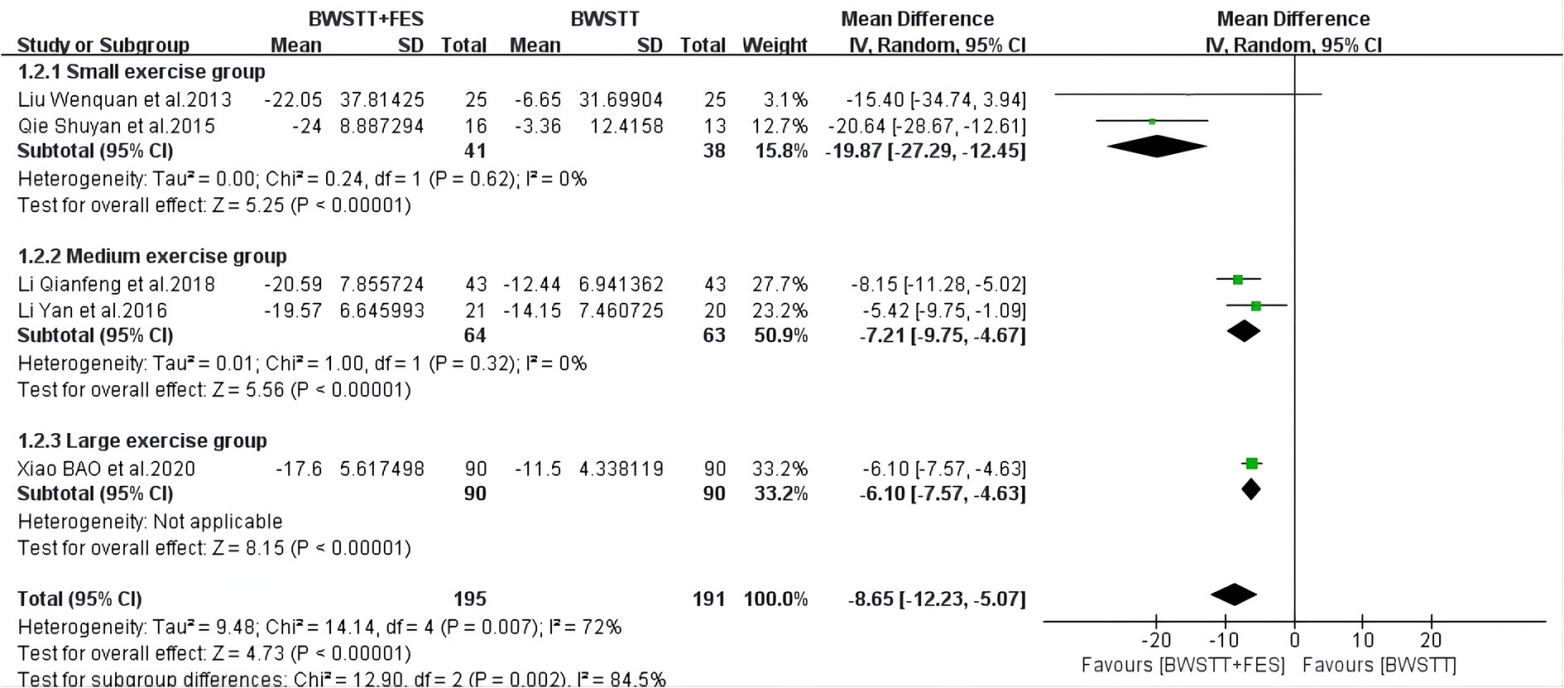
Forest plot showing the effect on 10 Meters Walking Test (10MWT) of body weight support treadmill training combined with functional electrical stimulation (BWSTT+FES) vs. body weight support treadmill training (BWSTT). CI, confidence interval; SD, standard deviation.

**Figure 5:**
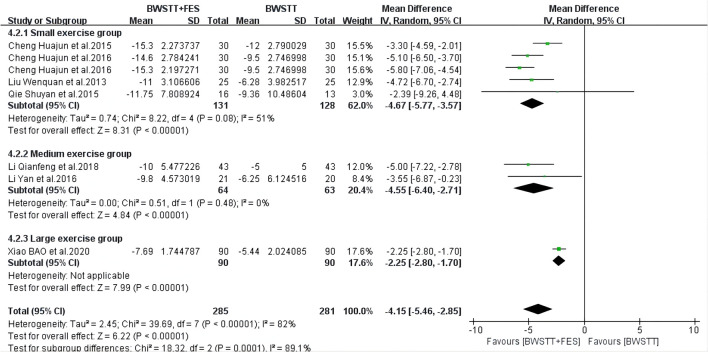
Forest plot showing the effect on Fugl-Meyer Assessment (FMA) of body weight support treadmill training combined with functional electrical stimulation (BWSTT+FES) vs. body weight support treadmill training (BWSTT). CI, confidence interval; SD, standard deviation.

Five of the 14 studies involved 10MWT indicators. When compared to BWSTT only, BWSTT combined with FES significantly improved 10MWT in stroke patients in the small exercise group (*I*^2^ = 0%; *p* < 0.00001), the medium exercise group (*I*^2^ = 0%; *p* < 0.00001), and the large exercise group (Figure 4). The effect of FMA on the small exercise group, the medium exercise group, and the large exercise group was similar to that of 10MWT (Figure 5). When compared to BWSTT only, BWSTT combined with FES had more significant effects on improving FMA in stroke patients.

**Figure 6:**
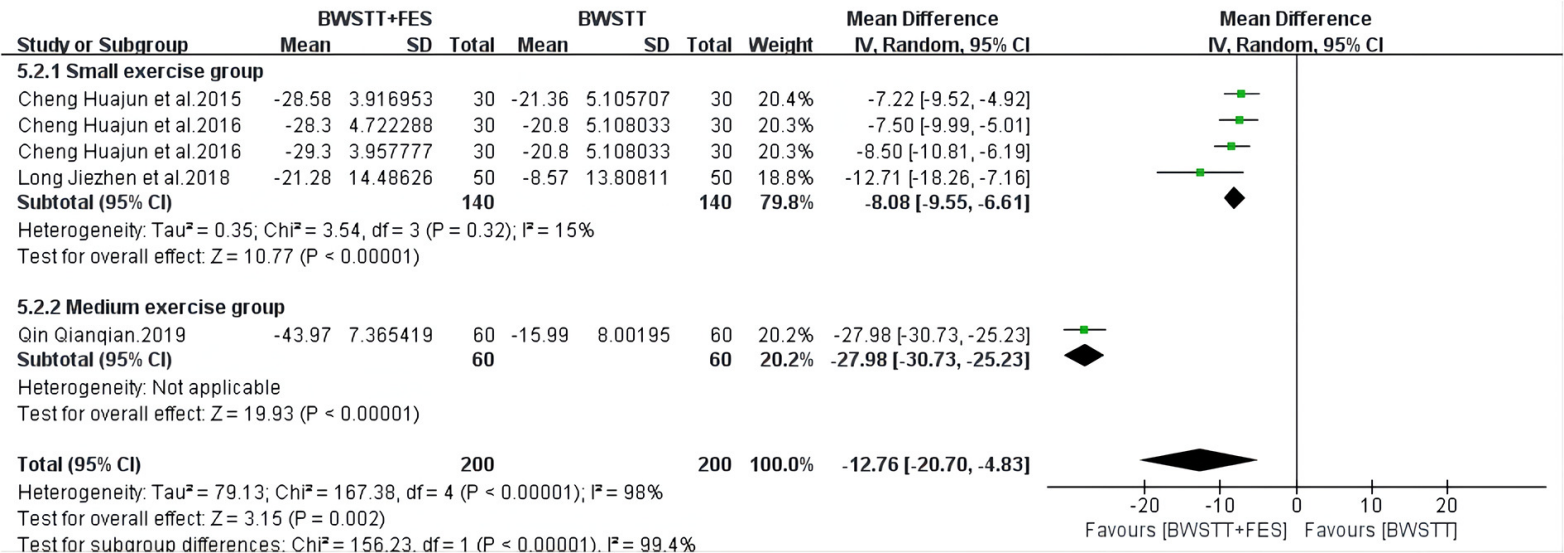
Forest plot showing the effect on Modified Barthel Index (MBI) of body weight support treadmill training combined with functional electrical stimulation (BWSTT+FES) vs. body weight support treadmill training (BWSTT). CI, confidence interval; SD, standard deviation.

**Figure 7:**
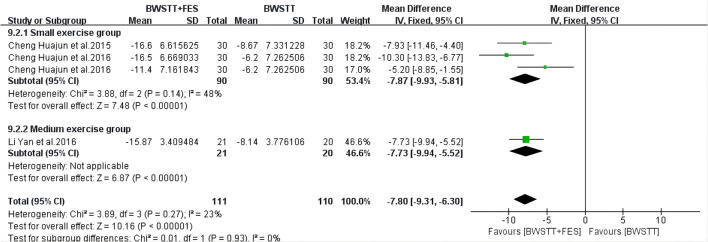
Forest plot showing the effect on Ankle Range of Motion (AROM) of body weight support treadmill training combined with functional electrical stimulation (BWSTT+FES) vs. body weight support treadmill training (BWSTT). CI, confidence interval; SD, standard deviation.

**Figure 8:**
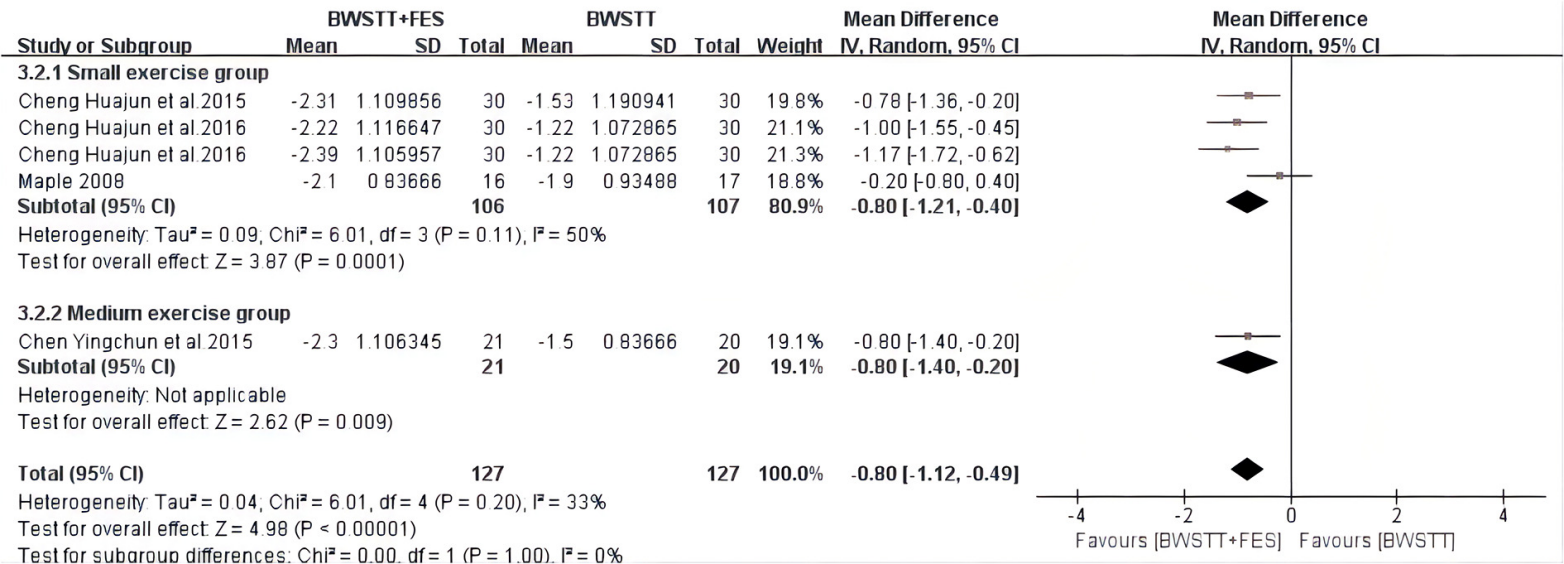
Forest plot showing the effect on Functional Ambulation Category (FAC) of body weight support treadmill training combined with functional electrical stimulation (BWSTT+FES) vs. body weight support treadmill training (BWSTT). CI, confidence interval; SD, standard deviation.

BWSTT combined with FES had a better effect on the MBI (*I*^2^ = 15%; *p* < 0.00001) (Figure 6), AROM (*I*^2^ = 48%; *p* < 0.00001) (Figure 7), and FAC (*I*^2^ = 50%; *p* = 0.0001) (Figure 8) of the small and medium exercise groups than BWSTT only.

**Figure 9:**
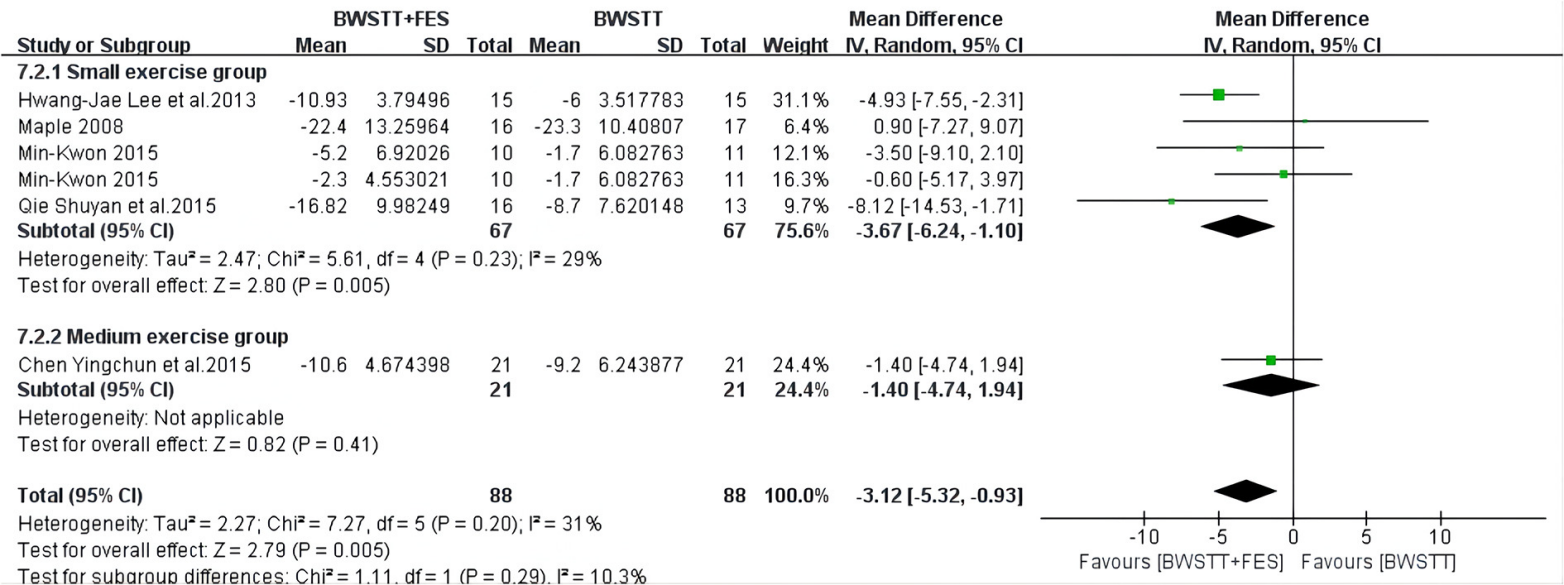
Forest plot showing the effect on Berg Balance Scale (BBS) of body weight support treadmill training combined with functional electrical stimulation (BWSTT+FES) vs. body weight support treadmill training (BWSTT). CI, confidence interval; SD, standard deviation.

**Figure 10:**
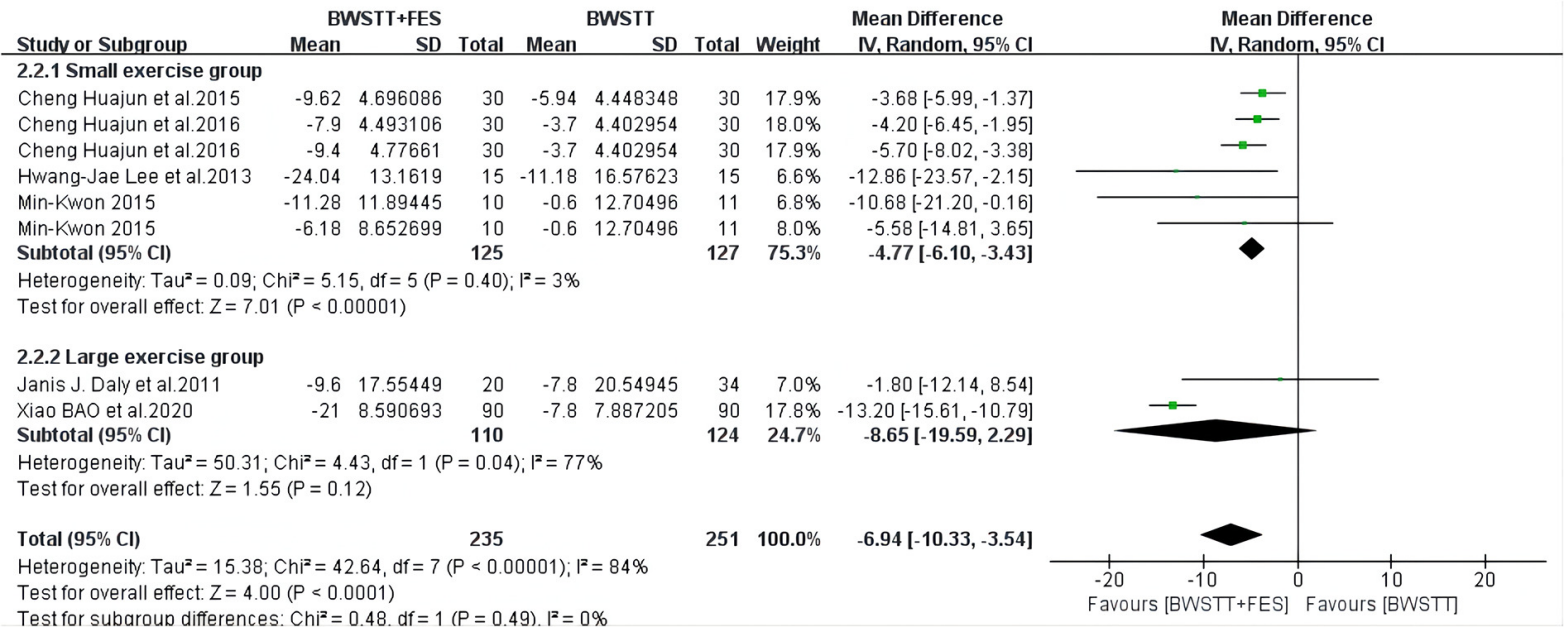
Forest plot showing the effect on gait speed of body weight support treadmill training combined with functional electrical stimulation (BWSTT+FES) vs. body weight support treadmill training (BWSTT). CI, confidence interval; SD, standard deviation.

When compared to BWSTT only, BWSTT combined with FES had a significant effect on the BBS (*I*^2^ = 29%; *p* = 0.005) (Figure 9) and gait speed (*I*^2^ = 0%; *p* < 0.00001) (Figure 10) of the small exercise group. However, when compared to BWSTT only, the effect of BWSTT combined with FES on the BBS of the medium exercise group was not significant. The effect of BWSTT combined with FES on the gait speed of the large exercise group was also not significant.

**Figure 11:**
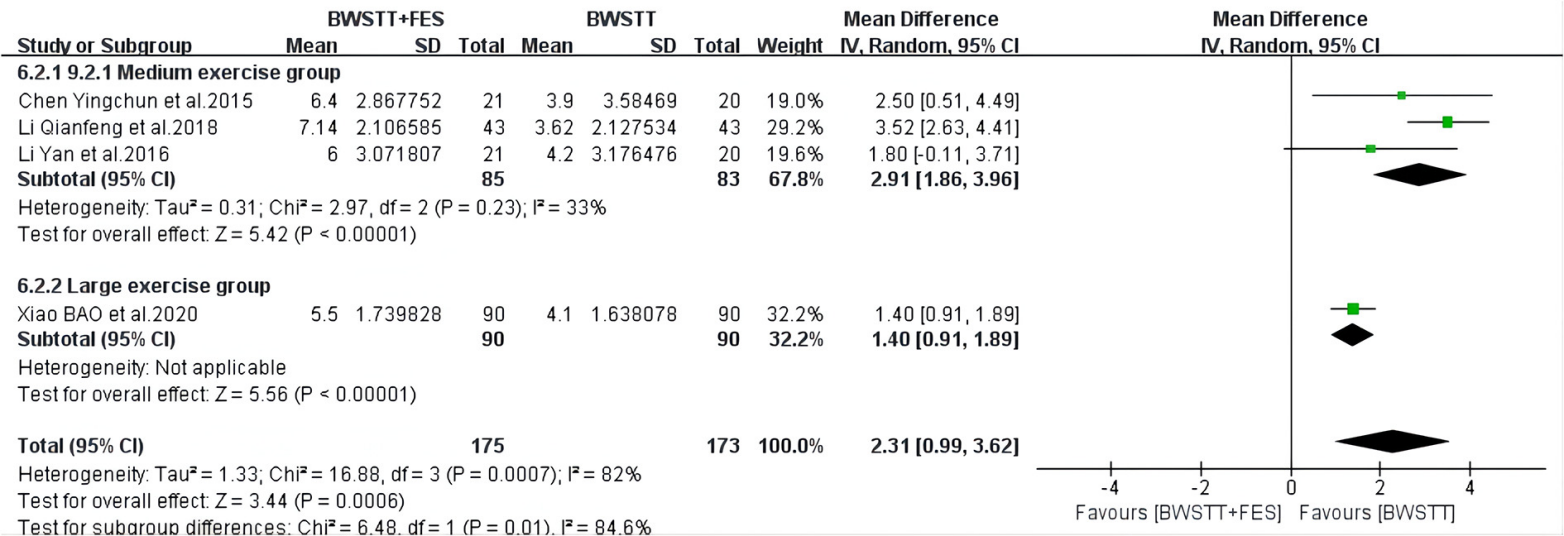
Forest plot showing the effect on Comprehensive Spasticity Scale (CSS) of body weight support treadmill training combined with functional electrical stimulation (BWSTT+FES) vs. body weight support treadmill training (BWSTT). CI, confidence interval; SD, standard deviation.

The effect of BWSTT combined with FES on CSS in the medium and large exercise groups (*I*^2^ = 33%; *p* < 0.00001) (Figure 11) was more significant than that of BWSTT only. CSS represents a comprehensive spasticity scale. Therefore, the smaller the value is, the less spasticity the stroke patients have; that is, the better the intervention effect is. Therefore, BWSTT combined with FES had a better improvement effect on the CSS of the medium and large exercise groups, when compared to BWSTT only.

### Sensitivity analysis

**Figure 12:**
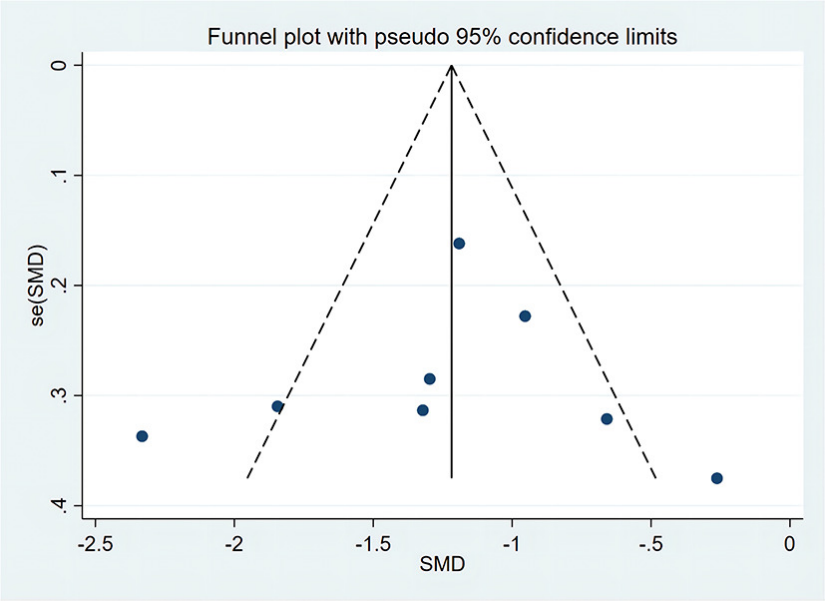
Funnel chart of body weight support treadmill training combined with functional electrical stimulation intervention and body weight support treadmill training in Fugl-Meyer Assessment (FMA).

We used Stata 17.0 to create funnel plots of each study index and evaluate possible publication bias. We then used Begg's test and Egger's test to evaluate the symmetry of the funnel plots, and defined *P* < 0.05 as significant publication bias. Finally, we used the cut-and-fill method to evaluate the effect of publication bias on the results. Meta-analysis showed that there was heterogeneity in the FMA, gait speed, and FAC, so sensitivity analysis was performed. The Egger's test revealed that the *P*-values of all the above indexes were > 0.05, (FMA *p* = 0.657 > 0.05, Figure 12), which indicated that there was no publication bias. Therefore, it was concluded that there was heterogeneity in the small number of included studies.

## Discussion

In this study, we compared the effects of BWSTT combined with FES and BWSTT only on the gait parameters of stroke patients. We found that BWSTT combined with FES has a better effect on the FAC, AROM, 10MWT, CSS, MBI, FMA, gait speed, and BBS of stroke patients, when compared to BWSTT only. However, there was no significant difference between the effects of BWSTT combined with FES and BWSTT only in improving the BBS of stroke patients in the medium exercise group. Also, there was no significant difference between the effects of BWSTT combined with FES and BWSTT only in improving the gait speed of stroke patients in the large exercise group.

BWSTT is a form of treatment that can effectively improve the walking ability and gait of stroke patients (21). In clinical and laboratory studies, BWSTT is usually used to promote the gait parameters of stroke patients. However, relevant studies showed that although BWSTT could improve the weight-bearing capability and self-balance of hemiplegic patients, its effect on improving the walking pattern was not good (31). Li et al. (17) reported that patients may experience lower limb spasms, droop, and varus during BWSTT training. FES can effectively alleviate the adverse effects of BWSTT. It can delay the occurrence and degree of lower limb spasms in hemiplegic patients at the early stage of stroke, and improve lower limb movement ability (32). FES has been proven to have played an important role in improving patients' gait parameters, knee coordination, and foot drop (23, 33). In recent years, the role of FES in the correction of foot droop and varus has also been partially confirmed (33, 34). It has been proven that, if patients are given the correct movement pattern to stimulate and improve proprioception, it would help in the reconstruction of the nervous system track. This can in turn improve patients' motor functions, and also play a key role in regulating their psychological state, metabolism, and immune mechanism (35, 36). In addition, in case of muscle denervation, FES could help maintain some level of muscle strength and muscle volume (37).

In the support phase, the quadriceps femores of hemiplegic patients are stimulated to promote weight-bearing, stimulate the contraction of the tibialis anterior to produce a reciprocal inhibitory effect, inhibit the spasm of the flexor digitorum muscle, and improve varus and foot drop. In the swing phase, the tibialis anterior is stimulated to induce ankle dorsiflexion, so as to facilitate foot clearance, thereby reducing the energy expended in walking and improving the patients' gait parameters (38, 39). Stimulation of the tibialis anterior through FES could also promote ankle dorsiflexion and improve ankle stability, which is very important for the recovery of gait parameters in stroke patients (40). Our meta-analysis showed that BWSTT combined with FES had a better effect on the gait parameters of stroke patients than BWSTT only. This may be due to the repair effect that FES has on damaged nerves, leading to an improvement in nerve conduction and neuromuscular excitability (41, 42). FES can aid the rehabilitation and treatment of post-stroke motor dysfunction by inducing the reactivation of paralyzed muscles, increasing the input of motor and sensory information, stimulating afferent nerves, stimulating the cortical sensory area with repetitive movement pattern information, forming excitation traces in the cortex, and awakening used neural pathways and synapses (43). This was also confirmed in our research results. When compared to BWSTT only, BWSTT combined with FES was indeed more effective in improving the gait parameters of stroke patients. This intervention could better improve the BBS, FAC, FMA, CSS, AROM, gait speed, MBI, and 10MWT of stroke patients, which is also consistent with previous research results (25, 28, 44).

Through subgroup analysis, we found that the effect of BWSTT combined with FES on the BBS of the medium exercise group was not significant when compared to that of BWSTT only. The analysis also showed that the effect of BWSTT combined with FES on the gait speed of the large exercise group was not significant when compared to that of BWSTT only. This result is similar to that reported by previous studies (45). Their results showed that there was no difference in performance between stroke patients who were given conventional overground gait training and those who used an electromechanical trainer with or without FES. This may be because FES can have an immune effect on gait speed and BBS after long-term exercise. The improvement effect was not significant in stroke patients, so there was no difference in the BBS and gait speed between BWSTT combined with FES and BWSTT only. In the small exercise group that was given BWSTT combined with FES, the stroke patients were more sensitively responsive to FES. Thus, the effect of BWSTT combined with FES was found to be more significant than BWSTT only. In addition, the gait speed and balance ability of stroke patients in the small exercise group were more easily improved by BWSTT combined with FES. In the long term, it was difficult to further improve the gait speed and balance ability of stroke patients who were given medium and large volume exercise. So, there was no difference between the effects of BWSTT only and BWSTT combined with FES on the BBS and gait speed of the medium and large exercise groups.

The limitations of this study are as follows. Firstly, this study was limited to published literature, and so publication bias cannot be entirely excluded. Secondly, there is potential heterogeneity in various factors such as the time and intensity of intervention, time and type of stroke, previous treatment, and duration between the stroke and the onset of its effects on the participants. Moreover, the duration of the intervention was short and inconsistent. The sample size of the study was small and most of the studies did not indicate whether they were selected randomly. Further, this meta-analysis and systematic review were limited in terms of inclusion criteria. That said, subgroup analyses were performed for all the gait parameters indexes, and descriptive analyses were performed for those not suitable for subgroup analysis. Sensitivity analysis was performed for the indexes with heterogeneity, so as to ensure a more comprehensive and accurate assessment.

## Conclusion

In this study, we compared the improvement in the gait parameters of stroke patients in small, medium, and large exercise groups of BWSTT combined with FES and BWSTT only, through systematic review and meta-analysis. When compared to BWSTT only, BWSTT combined with FES was observed to have a better effect on FAC, AROM, 10MWT, CSS, MBI, FMA, gait speed, and BBS. However, when compared to BWSTT only, the effect of BWSTT combined with FES on the BBS of the medium exercise group was not significant. In addition, when compared to BWSTT only, BWSTT combined with FES did not have a significant effect on the gait speed of the large exercise group. In general, when compared to BWSTT only, BWSTT combined with FES was found to be a more effective method for improving the gait parameters in stroke patients, and hence worthy of clinical promotion and application.

## Data availability statement

The original contributions presented in the study are included in the article/Supplementary material, further inquiries can be directed to the corresponding author/s.

## Author contributions

JW: literature search, research selection, quality evaluation, data analysis, and writing—original draft preparation. LZ and CL: literature search, research selection, and quality evaluation. YG: data extraction, data processing, writing—review and editing, and funding acquisition. XD: writing—review. XH: data extraction. All authors contributed to the article and approved the submitted version.

## Funding

This research was funded by the National Social Science Foundation of China (Grant Number: 21BTY054).

## Conflict of interest

The authors declare that the research was conducted in the absence of any commercial or financial relationships that could be construed as a potential conflict of interest.

## Publisher's note

All claims expressed in this article are solely those of the authors and do not necessarily represent those of their affiliated organizations, or those of the publisher, the editors and the reviewers. Any product that may be evaluated in this article, or claim that may be made by its manufacturer, is not guaranteed or endorsed by the publisher.

## Abbreviations

BWSTT: Body weight support treadmill training
FES: Functional electrical stimulation
10MWT: 10 Meters Walking Test
FMA: Fugl-Meyer Assessment
BBS: Berg Balance Scale
MBI: Modified Barthel Index
CSS: Comprehensive Spasticity Scale
FAC: Functional Ambulation Category
AROM: Ankle Range of Motion
CT: Computer Tomography
MRI: Magnetic Resonance Imaging
CI: Confidence Interval
SD: Standard Deviation.
