# Supplementary material for: The difference between the effectiveness of body-weight-supported treadmill training combined with functional electrical stimulation and sole body-weight-supported treadmill training for improving gait parameters in stroke patients: A systematic review and meta-analysis
Source: Front Neurol. 2022 Nov 9;13:1003723. doi: 10.3389/fneur.2022.1003723 (PMC9682182; doi:10.3389/fneur.2022.1003723)
Supplement: Supplementary file 1 [file Data_Sheet_1.docx]

**Search Strategy**

**Web of Science**

TS =(( "gait training" OR "walking training" OR "treadmill training" OR "body-weight support treadmill training" OR "functional electrical stimulation") AND ( stroke OR "brain attack" OR "brain injure" OR "brain injury" OR "brain infarct" OR "brain infection" OR "brain ischemia" OR "brain thrombus" OR "brain apoplexy" OR cerebral OR "cerebellar infarction" OR "cerebrovascular accident" OR "cerebrovascular accidents" OR CVA OR "cerebrovascular diseases" OR "cerebrovascular disorder" OR "ischemic cerebrovascular disease" OR hemiplegia OR hemiparesis OR "hemorrhage apoplexy" OR hematencephalon OR "intracerebral hemorrhage" OR ICH OR "brain hemorrhage" OR encephalorrhagia OR "subarachnoid hemorrhage" OR SAH OR "subarachnoidal bleeding" OR "Traumatic subarachnoid haemorrhage" OR paresis OR paretic))

**Pubmed**

("gait training" OR "walking training" OR "treadmill training" OR "body-weight support treadmill training" OR "functional electrical stimulation") AND (stroke OR "brain attack" OR "brain injure" OR "brain injury" OR "brain infarct" OR "brain infection" OR "brain ischemia" OR "brain thrombus" OR "brain apoplexy" OR cerebral OR "cerebellar infarction" OR "cerebrovascular accident" OR "cerebrovascular accidents" OR CVA OR "cerebrovascular diseases" OR "cerebrovascular disorder" OR "ischemic cerebrovascular disease" OR hemiplegia OR hemiparesis OR "hemorrhage apoplexy" OR hematencephalon OR "intracerebral hemorrhage" OR ICH OR "brain hemorrhage" OR encephalorrhagia OR "subarachnoid hemorrhage" OR SAH OR "subarachnoidal bleeding" OR "Traumatic subarachnoid haemorrhage" OR paresis OR paretic)

**Cochrane Library**

("gait training" OR "walking training" OR "treadmill training" OR "body-weight support treadmill training" OR "functional electrical stimulation" ) AND (stroke OR "brain attack" OR "brain injure" OR "brain injury" OR "brain infarct" OR "brain infection" OR "brain ischemia" OR "brain thrombus" OR "brain apoplexy" OR cerebral OR "cerebellar infarction" OR "cerebrovascular accident" OR "cerebrovascular accidents" OR CVA OR "cerebrovascular diseases" OR "cerebrovascular disorder" OR "ischemic cerebrovascular disease" OR hemiplegia OR hemiparesis OR "hemorrhage apoplexy" OR hematencephalon OR "intracerebral hemorrhage" OR ICH OR "brain hemorrhage" OR encephalorrhagia OR "subarachnoid hemorrhage" OR SAH OR "subarachnoidal bleeding" OR "Traumatic subarachnoid haemorrhage" OR paresis OR paretic)

**MEDLINE Ovid**

("gait training" OR "walking training" OR "treadmill training" OR "body-weight support treadmill training" OR "functional electrical stimulation") AND (stroke OR "brain attack" OR "brain injure" OR "brain injury" OR "brain infarct" OR "brain infection" OR "brain ischemia" OR "brain thrombus" OR "brain apoplexy" OR cerebral OR "cerebellar infarction" OR "cerebrovascular accident" OR "cerebrovascular accidents" OR CVA OR "cerebrovascular diseases" OR "cerebrovascular disorder" OR "ischemic cerebrovascular disease" OR hemiplegia OR hemiparesis OR "hemorrhage apoplexy" OR hematencephalon OR "intracerebral hemorrhage" OR ICH OR "brain hemorrhage" OR encephalorrhagia OR "subarachnoid hemorrhage" OR SAH OR "subarachnoidal bleeding" OR "Traumatic subarachnoid haemorrhage" OR paresis OR paretic)

**CNKI**

脑卒中 + 中风 + 卒中 + 偏瘫 + 出血性脑卒中 + 脑出血 + 蛛网膜下腔出血 + 缺血性脑卒中 + 脑缺血 + 脑血栓 + 脑栓塞 + 脑梗塞 + 脑梗死

**AND**步态训练 + 步行训练 + 步伐训练 + 步法训练 + 步态练习 + 跑步机训练 + 体重支持的跑步机步态训练 + 功能性电刺激 + 电刺激

**Wanfang data**

脑卒中 or 中风 or 卒中 or 偏瘫 or 出血性脑卒中 or 脑出血 or 蛛网膜下腔出血 or 缺血性脑卒中 or 脑缺血 or 脑血栓 or 脑栓塞 or 脑梗塞 or 脑梗死

步态训练 or 步行训练 or 步伐训练 or 步法训练 or 步态练习 or 跑步机训练 or 体重支持的跑步机步态训练 or 功能性电刺激 or 电刺激

**VIP**

M=((脑卒中 OR 中风 OR 卒中 OR 偏瘫 OR 出血性脑卒中 OR 脑出血 OR 蛛网膜下腔出血 OR 缺血性脑卒中 OR 脑缺血 OR 脑血栓 OR 脑栓塞 OR 脑梗塞 OR 脑梗死) AND (步态训练 OR 步行训练 OR 步伐训练 OR 步法训练 OR 步态练习 OR 跑步机训练 OR 体重支持的跑步机步态训练 OR 功能性电刺激 OR 电刺激))
